# ﻿Morphological and phylogenetic analyses reveal Xenosporidesmiaceae fam. nov, with seven *Xenosporidesmium* species

**DOI:** 10.3897/mycokeys.123.164006

**Published:** 2025-10-13

**Authors:** Ling-Ling Liu, Xiao-Feng Gu, Jiu-Lan Gou, Zuo-Yi Liu, Ya-Ya Chen, Jia-Hai Wu, Yong-Xiang Liu

**Affiliations:** 1 Guizhou Provincial Institute of Soil and Fertilizer, Guizhou Academy of Agricultural Sciences, Guiyang 550025, China Guizhou Academy of Agricultural Sciences Guiyang China; 2 Guizhou Provincial Key Laboratory of Agricultural Microbiology, Guizhou Academy of Agricultural Sciences, Guiyang 550025, China Guizhou Academy of Agricultural Sciences Guiyang China; 3 Guizhou Academy of Agricultural Sciences, Guiyang 550025, China Guizhou Academy of Agricultural Sciences Guiyang China; 4 Guizhou Provincial Institute of Crop Germplasm Resources, Guizhou Academy of Agricultural Sciences, Guiyang 550025, China Guizhou Academy of Agricultural Sciences Guiyang China

**Keywords:** 3 new taxa, asexual morph, phylogeny, Sordariomycetes, taxonomy

## Abstract

This study introduces a new family, Xenosporidesmiaceae, and describes a novel genus, *Xenosporidesmium*, along with a new species, *Xenosporidesmium caohaiense*, based on morphological and phylogenetic analyses. Multi-locus phylogenetic reconstruction (LSU, SSU, ITS, and *rpb*2) revealed that the new family forms a distinct clade, diverging from Sporidesmiales and Distoseptisporales in Sordariomycetes, and supporting its taxonomic independence. The new species, *X. caohaiense*, is characterized by dark brown conidiophores and pale brown, distoseptate conidia with mucilaginous sheaths. Additionally, six previously described *Sporidesmium* species are reclassified into *Xenosporidesmium*. This study emphasizes the significance of molecular data in resolving taxonomic ambiguities among morphologically similar but phylogenetically divergent fungi, thereby contributing to the understanding of freshwater fungal diversity.

## ﻿Introduction

*Sporidesmium* is a common dematiaceous hyphomycetous genus, which is generally recognized as having holoblastic phragmoconidia borne on simple, determinate, or percurrent conidiophores (Ellis 1958; 1971). Since the genus was introduced by Link (1809), numerous taxa have been accommodated to *Sporidesmium*. In a total of 504 epithets of *Sporidesmium* listed in Index Fungorum (August 2025), about 83% of them were proposed before 1992. Based on the diagnostic features such as euseptation/distoseptation, percurrent proliferation, and the absence or presence of conidiophores, Subramanian (1992) reassessed *Sporidesmium* into several genera. Species that have present conidiophores with none or percurrent and irregular proliferations, and euseptate conidia were disposed in *Sporidesmium* sensu stricto. Other genera, such as *Repetophragma* with annellate conidiogenous cells bearing euseptate conidia on present conidiophores, and *Stanjehughesia* with absent conidiophores and euseptate conidia produced on simple conidiogenous cells, were proposed (Subramanian 1992). Following Subramanian’s delimitation, McKenzie (1995) treated thirteen species of sporidesmium-like taxa, including three new species in *Ellisembia* and one in *Sporidesmium*. Hernández-Gutiérrez and Sutton (1997) introduced two genera, *Imimyces* (= *Imicles*) (Shoemaker and Hambleton 2001) based on distoseptate conidia on lageniform, doliiform, or ovoid proliferating conidiogenous cells, and *Linkosia* based on absent conidiophores and solitary and distoseptate conidia on simple conidiogenous cells. Wu and Zhuang (2005) merged *Imicles* into *Ellisembia* and *Penzigomyces* into *Sporidesmium*. The authors also established the genus *Novozymia* based on its annellidically proliferating conidiogenous cells, which differ from those of *Neosporidesmium*.

However, Subramanian’s delimitation criteria for *Sporidesmiumsensu lato* were rejected by Réblová (1999), who discussed the known connections between sexual morphs and their sporidesmium-like asexual morphs, as well as the morphological differences observed when fungi grow in nature and in culture. For example, *Stanjehughesia larvata* (= *Sporidesmium larvatum*) (Subramanian 1992) produces macronematous conidiophores when growing in nature but has semi-macronematous or micronematous conidiophores when growing in culture (Réblová 1999). Shenoy et al. (2006) revealed that *Sporidesmium* and morphologically similar genera are not monophyletic, being distributed across two distinct fungal classes: Dothideomycetes (e.g., *Sporidesmiella fusiformis*and *Sporidesmium obclavatulum*) and Sordariomycetes (e.g., *Stanjehughesia vermiculata*and *Linkosia fusiformis*). Since then, the polyphyletic nature of sporidesmium-like taxa was demonstrated in many studies (Su et al. 2016; Yang et al. 2018; Liu et al. 2019; Luo et al. 2019).The order Sporidesmiales containing *Sporidesmiumsensu stricto* is now placed in Diaporthomycetidae, Sordariomycetes (Crous et al. 2018; Hyde et al. 2020; Dong et al. 2021). Currently, 192 *Sporidesmium* names are listed in Species Fungorum (as of August 2025), with others transferred to more than 50 genera.

During a survey on freshwater fungi in China, two sporidesmium-like taxa were collected and identified on the basis of morpho-phylogenetic evidence. One new family, Xenosporidesmiaceae, is established to accommodate *Xenosporidesmium* gen. nov. and seven species of *Xenosporidesmium*.

## ﻿Materials and methods

### ﻿Sampling collection, examination, and isolation

Submerged dead wood samples collected from freshwater in Guizhou Province, China, were placed in zip-lock plastic bags, tagged with the collection information (Rathnayaka et al. 2025), and transported to the laboratory. The samples were then incubated in sterile plastic containers lined with moistened filter paper at room temperature for seven days to promote fungal growth. Motic SMZ 168 Series (Motic, Xiamen, China) and Nikon SMZ-171 (Nikon, Tokyo, Japan) dissecting microscopes were used to observe the fungal colonies and fruiting bodies. Fungal structures were examined and photographed using a Nikon ECLIPSE 80i (Nikon, Tokyo, Japan) compound microscope fitted with a Canon 600D/70D (Canon, Tokyo, Japan) digital camera.

Single-spore isolations were performed according to the method described by Senanayake et al. (2020). Germinated conidia were then aseptically transferred to fresh potato dextrose agar (PDA) plates for further cultivation. Dried specimens were deposited in the Herbarium of Cryptogams, Kunming Institute of Botany, Academia Sinica (HKAS), Kunming, China, and the Herbarium of Guizhou Academy of Agriculture Sciences (GZAAS), Guiyang, China. Pure cultures were deposited at the Guizhou Culture Collection (GZCC), Guiyang, China. The names of the new taxa were registered in Index Fungorum (2025) and Fungal Names (https://nmdc.cn/fungalnames/registe) (Wang et al. 2022).

### ﻿DNA extraction, PCR amplification, and sequencing

Fresh fungal mycelia grown on potato dextrose agar (PDA) were scraped with a sterilized toothpick and transferred to a 1.5 ml microcentrifuge tube for genomic DNA extraction. Genomic DNA was extracted using the Biospin Fungus Genomic DNA Extraction Kit (BioFlux, China), following the manufacturer’s protocol. Four gene regions, LSU, SSU, ITS and *rpb*2 were amplified using the primer pairs LR0R/LR5, NS1/NS4, ITS5/ITS4and *rpb*2-5F/ *rpb*2-7cR, respectively (Liu et al. 1999; Vilgalys and Hester 1990; White et al. 1990). The PCR amplification reactions were performed in a 25 µL reaction volume, comprising 1 µL of DNA, 1 µL of each forward and reverse primer, and 22 µL of 1.1 × T3 Super PCR Mix (Qingke Biotech, Chongqing, China). The conditions for the polymerase chain reaction (PCR) correspond to the reaction conditions reported by Liu et al. (2019). The PCR products were detected by 1% agarose gel electrophoresis, and the sequencing results were provided by Beijing Tsingke Biotechnology Co., Ltd.

### ﻿Phylogenetic analyses

The sequences incorporated in this study were downloaded from GenBank (Table 1; https://www.ncbi.nlm.nih.gov/). The single gene datasets (LSU, SSU, ITS, and *rpb*2) were aligned using MAFFT v.7.473 (https://mafft.cbrc.jp/alignment/server/, (Katoh and Standley 2013)) and trimmed using trimAl.v1.2rev59 software (Capella-Gutierrez et al. 2009) with the ‘gappyout’ option. Multiple gene alignments were merged using SequenceMatrix 1.7.8 (Vaidya et al. 2011).

**Table 1.** Taxa used in this study and their GenBank accession numbers.

Species	Strain	Type	LSU	SSU	ITS	*rpb*2
*Acidothrix acidophila*	CBS 136259		MH877622	NA	NA	NA
*Acrodictys aquatic*	MFLUCC 18-0356		MG835712	NA	MG835711	NA
*Acrodictys bambusicola*	CGMCC 3.18641		KX033564	KX033535	KU999973	NA
*Acrodictys chishuiensis*	HKAS 112618	T	OP377909	OP377995	OP377810	OP473084
*Acrodictys effuse*	GZAAS 20-0446	T	OP377936	NA	MW133879	NA
*Afroraffaelea ambrosiae*	CBS 141678		NG_057115	NG_061251	NA	NA
*Amplistroma caroliniana*	BEO 9923		FJ532377	NA	NA	NA
*Annulatascus velatisporus*	MFLUCC 16-1441		KY244031	KY244032	NA	NA
*Annulatascus velatisporus*	HKUCC 3701	T	AF132320	NA	NA	NA
*Annulusmagnus triseptatus*	A54 10A		AY590286	NA	NA	NA
*Annulusmagnus triseptatus*	CBS 128831	T	GQ996540	JQ429242	NA	JQ429258
*Aquapteridospora lignicola*	MFLUCC 15-0377	T	KU221018	NA	NA	NA
*Aquapteridospora linzhiensis*	KUNCC 10420	T	OQ970576	NA	OP626343	NA
*Aquapteridospora hyaline*	GZCC 22-0072	T	ON527945	NA	ON527937	NA
*Ascitendus austriacus*	CBS 102665		NG_056942	NG_061014	NA	NA
*Ascotaiwania latericolla*	ICMP 22739		MN699407	NA	NR_173185	MN704312
*Atractospora reticulate*	CBS 127884		KT991660	NA	NA	KT991649
*Aureobasidium pullulans*	CBS 584.75		NG_055734	NG_062753	NR_144909	NA
*Barbatosphaeria barbirostris*	CBS 121149		EF577059	KM492851	NA	KM492903
*Barbatosphaeria dryina*	CBS 127691		KM492864	KM492852	KM492890	KM492904
*Barbatosphaeria neglecta*	CBS 127693		KM492868	KM492856	NA	NA
*Barretomyces calatheae*	CBS 129274		KM484950	NA	KM484831	NA
*Brachysporium nigrum*	MR 1346		KT991662	KT991643	NA	KT991652
*Bullimyces communis*	AF281.5		JF775587	JF758619	NA	NA
*Bussabanomyces longisporus*	CBS 125232		KM484951	NG_061183	KM484832	NA
*Calosphaeria pulchella*	CBS 115999		AY761075	AY761071	NA	GU180661
*Canalisporium caribense*	SS03839		GQ390268	GQ390253	GQ390283	NA
*Cancellidium atrobrunneum*	MFLUCC 20-0100		MT422740	MT422726	NA	NA
*Ceratolenta caudate*	CBS 125234		JX066704	NG_061136	NA	JX066699
*Chaetopsina pinicola*	CBS136444		NG_058865	NA	NA	NA
*Coniolariella gamsii*	CBS 117677		MH874576	NA	NA	NA
*Conlarium duplumascospora*	CGMCC 3.14938	T	JN936991	JN936987	NR_138382	NA
*Cryphonectria parasitica*	CMW 7084		JN940858	JN938760	JN942325	NA
*Cryptadelphia groenendalensis*	SMH 3767		EU528001	NA	NA	NA
*Cryptadelphia groenendalensis*	SH 12		EU528007	NA	NA	NA
*Cytospora thailandica*	MFLUCC 17-0263		NG_064536	NA	NA	MH253464
*Deightoniella roumeguerei*	CBS 128780		JF951176	NA	JF951153	NA
*Diaporthe eres*	AR3538		AF408350	NA	NA	NA
*Dictyosporella aquatic*	MD1302		KT241022	KT241023	NA	NA
*Distoseptispora aquatic*	MFLUCC 15-0374	T	KU376268	NA	MF077552	NA
*Distoseptispora fluminicola*	DLUCC 0391	T	MG979762	NA	MG979755	NA
*Distoseptispora fluminicola*	MFLUCC 15-0417	T	KU376270	NA	MF077553	NA
*Distoseptispora guttulata*	MFLUCC 16-0183	T	MF077554	MF077532	MF077543	NA
*Distoseptispora obpyriformis*	MFLUCC 17-1694	T	MG979764	NA	NA	MG988415
*Distoseptispora rostrata*	MFLUCC 16-0969	T	MG979766	NA	MG979758	MG988417
*Distoseptispora tectonae*	MFLUCC 12-0291	T	KX751713	NA	KX751711	KX751708
*Dothidea hippophaeos*	CBS 188.58		DQ678048	NA	MH857750	NA
*Dothidea insculpta*	CBS 189.58		MH869284	DQ247810	NA	NA
*Flabellascus tenuirostris*	CBS 138680		NG_058200	NA	NR_136138	NA
*Flabellascus tenuirostris*	CBS 138690		KT716458	NA	KT716467	NA
*Fluminicola saprophytica*	MFLUCC 15-0976		MF374367	MF374375	NA	MF370954
*Fragosphaeria purpurea*	CBS 133.34		AB189154	AF096176	NA	NA
*Gnomoniopsis chamaemori*	CBS 803.79		EU255107	NA	NA	NA
*Halazoon melhae*	MF819		GU252143	GU252144	NA	NA
*Harknessia eucalypti*	CPC 13643		JQ706215	NA	NA	NA
*Hawksworthiomyces taylorii*	CMW 20741	T	NG_059701	NA	NR_155176	NA
*Hydea pygmea*	NBRC33069		GU252133	GU252134	NA	NA
*Jattaea prunicola*	STEU 6201		EU367456	EU367462		NA
*Jennwenomyces navicularis*	BCRC FU30872		MT224909	NA	MT224914	NA
*Jobellisia fraternal*	SMH 2863		AY346285	NA	NA	NA
*Jobellisia guangdongensis*	HMAS 251240		NG_068733	NA	NR_138381	NA
*Jobellisia guangdongensis*	DQ09		MN733255	NA	MN733257	NA
*Jobellisia luteola*	SMH 2753		AY346286	NA	NA	NA
*Junewangia sphaerospora*	CGMCC 3.18655		KX033572	KX033543	KU999981	NA
*Kohlmeyeriopsis medullaris*	CBS 117849		KM484968	NA	KM484852	NA
*Lanspora coronate*	AFTOL-ID 736		U46889	NA	NA	DQ470899
*Lulworthia fucicola*	ATCC 64288		AY878965	AY879007	NA	NA
*Magnaporthe salvinii*	M 21		JF414887	JF414862	NA	NA
*Myrmecridium iridis*	CPC 25084		KR476777	NA	KR476744	NA
*Nakataea oryzae*	CBS 252.34		MH867001	NA	KM484862	NA
*Natarajania thailandica*	MFLUCC 18-0394		MT371074	NA	NA	MT364367
*Neomyrmecridium guizhouense*	GZCC 20-0008		MT002307	NG_068427	NA	MT023016
*Neomyrmecridium septatum*	CBS 145073		NG_066289	NA	NR_161133	MK047544
*Neospadicoides lignicola*	MFLUCC 17-2444	T	MK849854	MK828309	MK828702	MN124532
*Ophiostoma piliferum*	AFTOL-ID 910		DQ470955	DQ471003	NA	DQ470905
*Papulosa amerospora*	AFTOL-ID 748		DQ470950	DQ470998	NA	DQ470901
*Pararamichloridium livistonae*	CPC 32156		MG386084	NA	NA	NA
*Phaeoacremonium chiangmaiense*	CMUB 40065	T	PQ699722	NA	PQ699720	NA
*Phaeoacremonium chiangmaiense*	CMU511		PQ699723	NA	PQ699721	NA
*Phaeoacremonium fraxinopennsylvanica*	MR 3064		HQ878595	HQ878600	NA	HQ878609
*Phaeoacremonium minimum*	CBS 6580		AY761082	AY761068	NA	DQ470913
*Phialemoniopsis ocularis*	UTHSC 05-2527		HE599266	NA	HE599281	NA
*Phomatospora bellaminuta*	AFTOL-ID 766		FJ176857	FJ176803	NA	FJ238345
*Pleurostoma ootheca*	CMU 23858		AY761079	AY761074	NA	HQ878606
*Polylobatispora deltoidea*	NBRC 106820		LC495605	NA	LC495612	NA
*Pseudoproboscispora thailandensis*	MFLUCC 15-0989		NG_059843	NG_063646		NA
*Pseudostanjehughesia aquitropica*	MFLUCC 16-0569		MF077559	MF077537	MF077548	NA
*Pyricularia grisea*	BR0029		KM484995	NA	KM484880	NA
*Pyriculariopsis parasitica*	HKUCC5562		DQ341514	NA	NA	NA
*Rhamphoria delicatula*	CBS 132724		MH878338	NA	NA	NA
*Rhodoveronaea varioseptata*	CBS 431.88		EU041870	NA	EU041813	NA
*Rubellisphaeria abscondita*	CBS 132078		KT991666	KT991646	NA	KT991657
*Savoryella lignicola*	NF00204		HQ446378	HQ446300	NA	NA
*Spadicoides bina*	CBS 137794		KY931824	KY931881	KY931796	KY931851
*Sporidesmiella hyalospermum*	MFLUCC 18-1312		MK849839	NA	MK828688	MN124520
*Sporidesmium appendiculatum*	MFLU 18-0981	T	MW287774	NA	NR_172451	NA
*Sporidesmium aquaticum*	MFLUCC 15-0420		KU376273	NA	NA	NA
*Sporidesmium bambusicola*	HKUCC 3578		DQ408562	NA	NA	NA
*Sporidesmium dalbergiae*	CPC 38960		MZ064481	NA	MZ064424	NA
*Sporidesmium diplazii*	KUNCC 23-13844	T	PQ671194	NA	PQ671275	PQ662531
*Sporidesmium dulongense*	MFLUCC 17-0116	T	NG_073631	NA	NR_171826	NA
*Sporidesmium minigelatinosa*	NN47497		DQ408567	NA	NA	DQ435090
*Sporidesmium pronephrii*	KUNCC 23-13909	T	PQ671195	NA	PQ671276	PQ662532
*Sporidesmium pyriformatum*	MFLUCC 15-0620		KX710141	NA	KX710146	MF135649
*Sporidesmium thailandense*	MFLUCC 15-0964		MF374370	NA	MF374361	MF370955
*Sporidesmium tratense*	MFLUCC 17-2392	T	OP377975	OP378050	OP377889	OP473120
*Sporidesmium tropicale*	MFLUCC 16-0185	T	MF077562	MF077539	MF077551	MF135646
*Sporidesmium tropicale*	HKUCC 10838		DQ408560	NA	NA	NA
*Striaticonidium cinctum*	CBS 932.69	T	KU847265	NA	KU847239	KU847290
*Synnemasporella toxicodendri*	CFCC 52097		MG682029	NA	MG682089	MG682049
*Thailandiomyces bisetulosus*	BCC00018		EF622230	EF622229	NA	NA
*Thyridium vestitum*	AFTOL-ID 172		AY544671	AY544715	NA	DQ470890
*Tirisporella beccariana*	BCC36738		JQ655451	JQ655453	NA	NA
*Togniniella acerosa*	ICMP 18256		AY761076	AY761073	NA	GU180660
*Volutella consors*	CBS 189.73		MH878487	NA	NA	NA
*Xenocylindrocladium serpens*	CBS 128439		MH876378	NA	NA	NA
*Xenosporidesmium aquaticivaginatum*	MFLUCC 15-0624		KX710142	MF077541	KX710147	MF135647
*Xenosporidesmium aquaticivaginatum*	KUNCC 23-13373		OR600972	OR743215	OR589324	OR820910
***Xenosporidesmium caohaiense***	**GZCC 19-0534**	**T**	**MZ227226**	**MW134698**	**MW133918**	**NA**
***Xenosporidesmium caohaiense***	**GZCC 19-0533**		**MZ227225**	NA	**MW133917**	**NA**
*Xenosporidesmium filiforme*	HKAS 130435	T	OR600973	OR743216	OR589325	OR820911
*Xenosporidesmium guizhouense*	CGMCC 3.19605	T	MK818586	MK818588	MK818587	MK828516
*Xenosporidesmium guttulatum*	KUNCC 23-13374	T	PP994882	OR743217	OR589326	OR820912
*Xenosporidesmium nujiangense*	DLUCC 983	T	NG_088244	MZ420748	NR_175741	MZ442695
*Xenosporidesmium olivaceoconidium*	MFLUCC 15-0380		KX710139	MF077542	KX710144	MF135648
*Xylaria hypoxylon*	AFTOL-ID 51		AY544648	NA	NA	DQ470878
*Xylolentia brunneola*	PRA 13611		MG600398	NA	MG600394	MG600402

Note: “T” indicates ex-type strains. Newly generated sequences are inbold. “NA” indicates the unavailable data in GenBank.

The maximum likelihood (ML) tree was conducted in the IQ Tree webserver (http://iqtree.cibiv.univie.ac.at/) (Nguyen et al. 2014) with 1,000 ultrafast bootstrap (BS) replicates. The substitution model option was set to auto. Maximum likelihood bootstrap supports (ML-BS) equal to or greater than 75% are marked above or below each branch.

Bayesian inferences (BI) were performed using a Markov chain Monte Carlo (MCMC) algorithm in MrBayes 3.2.6 (Ronquist et al. 2012). The best-fit substitution models for each single gene alignment were decided by MrModeltest 2.3 (Nylander 2008) under the Akaike Information Criterion (AIC). Two parallel runs of four simultaneous Markov chains were performed for 10,000,000 generations, or the searches were stopped when the average standard deviation of split frequencies was below 0.01 (stopval = 0.01). Trees were sampled every 1,000^th^ generations. The first 25% of trees were set as burn-in and discarded. The remaining trees were used to calculate posterior probabilities (BYPP). BYPP supports equal to or greater than 0.95 and is marked above or below each branch.

Phylogenetic trees were visualized using FigTree v1.4.4 and subsequently edited in Adobe Illustrator CC 2019 (v23.1.0; Adobe Systems, USA). Photo-plate and scale bars were prepared using Adobe Photoshop CC 2019 (Adobe Systems, USA) and the Tarosoft (R) Image Framework program, respectively.

## ﻿Phylogenetic results

**Figure 1.**
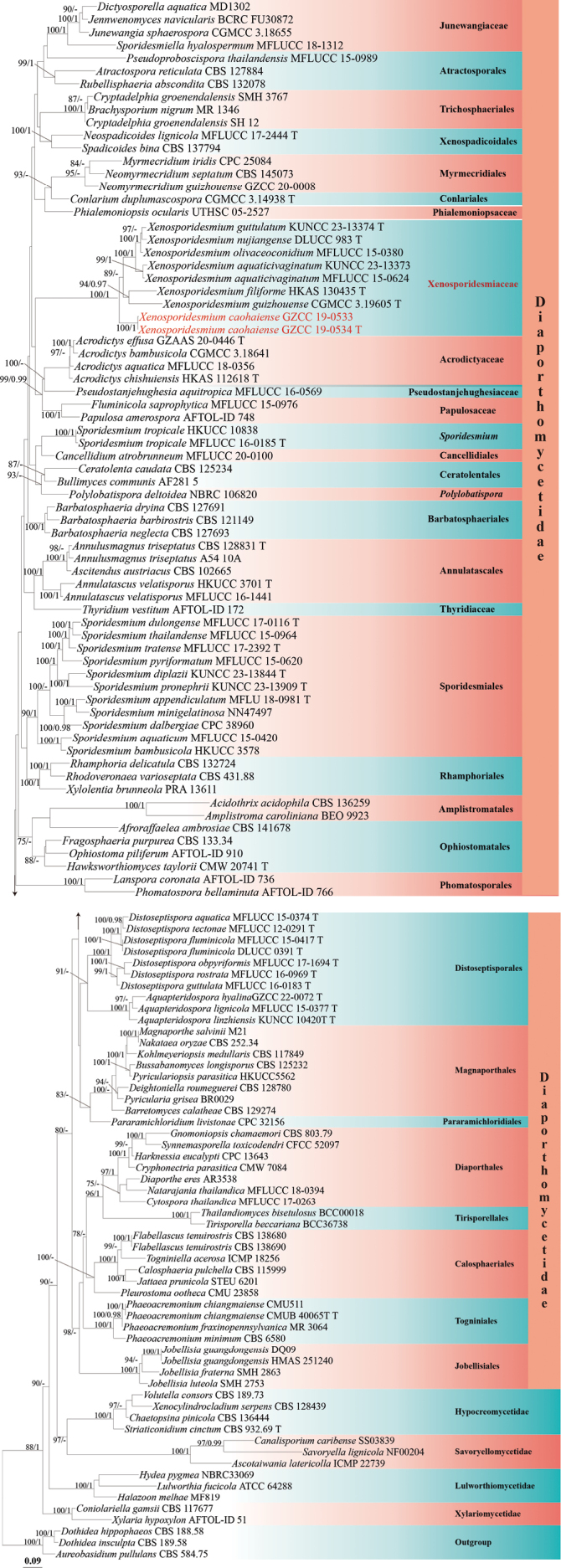
Phylogenetic tree generated from ML analysis based on the combined LSU, SSU, ITS, and *rpb*2 sequence data. Bootstrap support values for ML (≥ 75%) and BYPP (≥ 0.95) are indicated near their respective nodes. The tree is rooted with *Aureobasidium pullulans* CBS 584.75, *Dothidea hippophaeos* CBS 188.58, and *Dothidea insculpta* CBS 189.58. Newly obtained strains are in red.

The phylogenetic placement of the two newly obtained strains was determined through multi-locus phylogenetic analysis. The concatenated sequence matrix comprised 3,292 characters (LSU: 1–822, SSU: 823–1,800, ITS: 1,801–2,273, and *rpb*2: 2,274–3,292) across 127 taxa. Both ML and BYPP analyses produced congruent topologies. The best ML tree with a final likelihood value of -60836.732 is presented in Fig. 1.

Based on the multi-gene phylogenetic analysis (Fig. 1), our isolates (GZCC 19-0533 and GZCC 19-0534) represent a distinct species of *Xenosporidesmium*, forming a well-supported basal clade within the genus. Notably, this new species clusters with six other taxa—*X. aquaticivaginatum*, *X. filiforme*, *X. guizhouense*, *X. guttulatum*, *X. nujiangense*, and *X. olivaceoconidium*—to comprise a monophyletic lineage that is phylogenetically distinct from Sporidesmiales. Furthermore, this clade is phylogenetically sister to three *incertae sedis* families in Diaporthomycetidae (Acrodictyaceae, Papulosaceae and Pseudostanjehughesiaceae). Given the consistent molecular divergence and morphological uniqueness, we propose recognition of this clade as a new family, Xenosporidiaceae (Diaporthomycetidae, family *incertae sedis*).

### ﻿Taxonomy

#### ﻿ Xenosporidesmiaceae

Taxon classificationFungiDistoseptisporalesXenosporidesmiaceae

L. L. Liu & Z. Y. Liu fam. nov.

D226EE3E-473C-5938-8A9D-6604C9B929FB

Fungal Names: FN 572989

##### Etymology.

Referring to the name of the type genus.

##### Description.

***Colonies*** on nature substrate effuse, hairy, scattered or in small groups. ***Conidiophores*** macronematous, mononematous, solitary, cylindrical, straight or slightly flexuous, dark brown, septate. ***Conidiogenous cells*** holoblastic, monoblastic, integrated, terminal, cylindrical, smooth, pale to dark brown. ***Conidia*** acrogenous, solitary, obclavate, pale brown to olivaceous, paler at the apex, tapering gradually towards the apex, smooth, straight or curved, distoseptate, with a mucilaginous sheath or apical appendage.

##### Type genus.

*Xenosporidesmium* L. L. Liu & Z. Y. Liu, gen. nov.

##### Notes.

The new family Xenosporidesmiaceae is established to accommodate a distinct lineage comprising six *Sporidesmium* species and one new species described herein. This lineage forms a highly supported clade (ML/BS = 100%, BYPP = 1) in the multi-gene phylogeny (LSU, SSU, ITS, and *rpb*2), diverging from both Sporidesmiales and Distoseptisporales. All members share pale brown, sheathed or appendaged conidia and are exclusively associated with freshwater habitats (Hyde et al. 2016; Liu et al. 2019; Wang et al. 2024). The family is proposed to resolve the phylogenetic placement of these morphologically similar yet evolutionarily distinct taxa, and its establishment aims to avoid taxonomic confusion with the currently recognized *Sporidesmiumsensu stricto*.

#### ﻿ Xenosporidesmium

Taxon classificationFungiDistoseptisporalesXenosporidesmiaceae

L. L. Liu & Z. Y. Liu gen. nov.

2AA56D50-D3C9-56D3-AA13-AF0D12398DCD

Index Fungorum: IF904245

##### Etymology.

“Xeno-” means different, “*Xenosporidesmium*” reflects that, although this fungus resembles *Sporidesmium* in morphology, it is genetically distinct and phylogenetically separate.

##### Type species.

*Xenosporidesmium aquaticivaginatum* (Jiang Yang & K.D. Hyde) L. L. Liu & Z. Y. Liu, comb. nov.

##### Description.

***Saprobic*** on dead wood. **Sexual morph**: Undetermined. **Asexual morph**: ***Colonies*** on nature substrate effuse, hairy, scattered or in small groups. ***Conidiophores*** macronematous, mononematous, solitary, cylindrical, straight or slightly flexuous, dark brown, septate. ***Conidiogenous cells*** holoblastic, monoblastic, integrated, terminal, determinate or with one percurrent proliferation, cylindrical, smooth, pale to dark brown. ***Conidia*** acrogenous, solitary, obclavate, pale brown to olivaceous, paler at the apex, tapering gradually towards the apex, smooth, straight or curved, distoseptate, with a mucilaginous sheath or apical appendage.

##### Notes.

The new genus *Xenosporidesmium* is established to accommodate seven species, including one newly discovered taxon and six species previously classified in *Sporidesmium. Xenosporidesmium aquaticivaginatum* (=*Sporidesmium aquaticivaginatum*) is designated as the type species because its epithet reflects the aquatic habitat preference characteristic of this genus, although it was published concurrently with *X. olivaceoconidium* in the same work (Hyde et al. 2016). In this study, we introduce one new *Xenosporidesmium* species and transfer *Sporidesmium aquaticivaginatum*, *S. filiforme*, *S. guizhouense*, *S. guttulatum*, *S. nujiangens*, and *S. olivaceoconidium* to *Xenosporidesmium* based on phylogenetic analyses.

#### ﻿ Xenosporidesmium caohaiense

Taxon classificationFungiDistoseptisporalesXenosporidesmiaceae

L. L. Liu & Z. Y. Liu sp. nov.

F19475B5-3072-5237-99D9-BB39FEAA61C6

Index Fungorum: IF904247

Fig. 2

##### Etymology.

The epithet “caohaiense” refers to the type location, Caohai National Nature Reserve.

**Figure 2.**
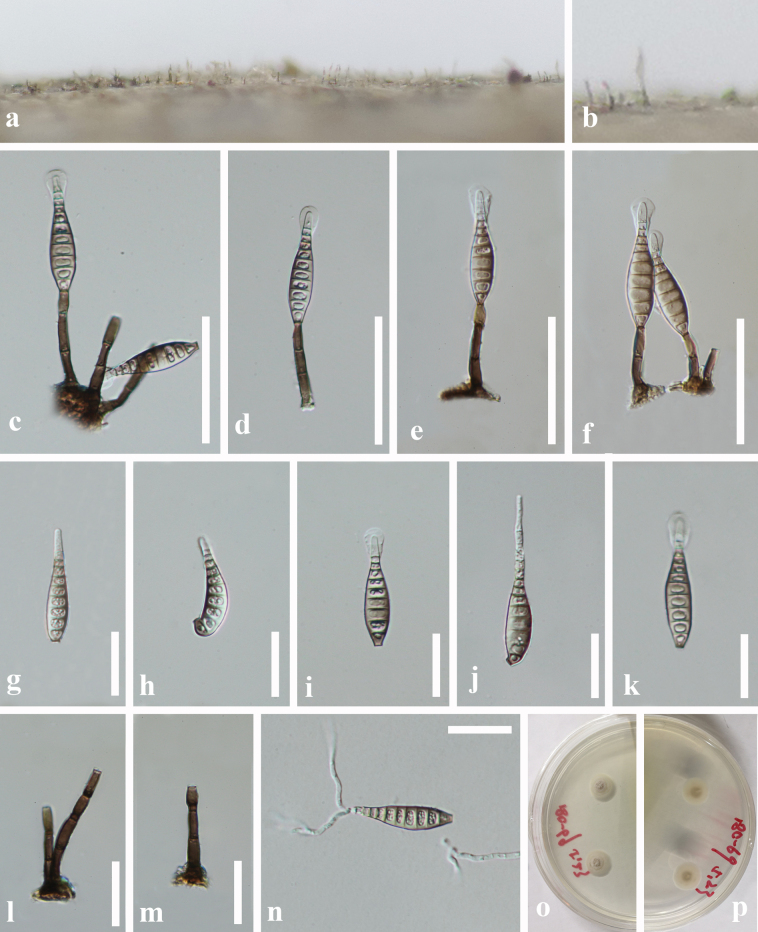
*Xenosporidesmium caohaiense* (HKAS 150269, holotype) a, b. Colony on wood; c–f. Conidiophores and conidia; g–k. Conidia; l, m. Conidiophores with conidiogenous cells; n. Germinated conidium; o, p. Culture; o. from above; p. from below. Scale bars: 50 μm (c–f, l, m); 25 μm (g–m, n).

##### Holotype.

HKAS 150269

##### Description.

***Saprobic*** on decaying submerged wood in freshwater habitats. **Sexual morph**: Unknown. **Asexual morph**: Hyphomycetous. ***Colonies*** on natural substrate effuse, hairy, dark brown, scattered or in small groups, velvety. ***Mycelium*** mostly immersed, composed of pale to brown, branched, septate, smooth hyphae. ***Conidiophores*** macronematous, mononematous, erect, solitary, straight or slightly flexuous, unbranched, cylindrical, dark brown, 1–3-septate, 25.5–50 × 3.5–6 μm (*x̄* = 37 × 4.5 μm, n = 20). ***Conidiogenous cells*** monoblastic, integrated, terminal, cylindrical or lageniform, pale brown, (6–)9–14.5 × 2.5–5.5 μm (*x̄* = 11 × 4 μm, n = 20). ***Conidia*** 35–51 × 7.5–11 μm (*x̄* = 42 × 9 μm, n = 20), acrogenous, solitary, obclavate, pale brown, straight or slightly flexuous, apically rostrate, hyaline to pale brown, often with a mucilaginous sheath, truncate at the base, 8–13-distoseptate, guttulate, smooth-walled, thin-walled.

##### Culture characteristics.

Conidia germinating on PDA medium within 12 h. Germ tubes produced from the apex. Colonies on PDA medium reaching 20 mm diam. after 3 weeks at 25 °C, circular, flat, centrally umbonate, with velvety, white to pale yellow mycelium on the surface; in reverse pale yellow with entire margin.

##### Material examined.

China • Guizhou Province, Bijie City, Weining District, Caohai National Nature Reserve, on decaying wood submerged in a freshwater lake, 4 October 2018, L.L. Liu, 18C-69 (HKAS 150269, holotype; GZAAS 20-0429, isotype), ex-type cultures GZCC19-0534; China • Guizhou Province, Zunyi City, Chishui District, on decaying wood submerged in a freshwater stream, 16 July 2019, J. Yang, CS2-6-1 (HKAS 150268), living cultures GZCC 19-0533.

##### Notes.

*Xenosporidesmium caohaiense* forms a distinct lineage and clusters within the *Xenosporidesmium* clade by high support (ML-BS = 100%, BYPP = 1) in the phylogenetic tree, indicating a close relationship with other *Xenosporidesmium*. The morphological differences with other *Xenosporidesmium* species are shown in Table 2. *Xenosporidesmium filiforme* is distinguished from other *Xenosporidesmium* species by possessing an apical appendage. *Xenosporidesmium caohaiense* can be differentiated from all other species (except *X. olivaceoconidium*) based on the dimensions of its conidiophores, conidiogenous cells,and conidia. Although *X. caohaiense* and *X. olivaceoconidium* exhibit highly similar morphological characteristics, they are phylogenetically distant in the tree. Additionally, there are 43 bp differences (313 bp, without gaps) in the ITS region between these two species. Therefore, we introduce *Xenosporidesmium caohaiense* as a novel species here.

**Table 2.** Comparative morphological characteristics of *Xenosporidesmium* species.

Taxa	Strain	Conidiophores	Conidiogenous cells	Conidia
*X. aquaticivaginatum*	MFLUCC 15-0624	60–125 × 4–6	NA	49.5–80.5 × 10.5–14,6–10-distoseptate, with a sheath
*X. caohaiense*	GZCC 19-0534	25.5–50 × 3.5–6	(6–)9–14.5 × 2.5–5.5	35–51 × 7.5–11, 8–13-distoseptate, with a sheath
*X. filiforme*	HKAS 130435	22–54 × 3.5–4.9 µm	12–16 × 3.6–4.9 µm	53–100 × 5.8–9.5 µm, mostly 10-distoseptate, with an apical appendage
*X. guizhouense*	CGMCC3.19605	23–59 × 2–4.6	13–25 × 3.1–4	46–86 × 7–11.4, 6– 10-septate, with a sheath
*X. guttulatum*	KUNCC 23-13374	26–55 × 3.4–5 µm	12– 20 × 3–4.8 µm	44–84 × 8.7–12 µm,6–14-distoseptate, with a sheath
*X. nujiangense*	DLUCC 983	31–51 × 4–5.5 µm	11.5–16.5 × 4–5 µm	54–69 × 10–12.5 µm, 10–14-septate, with a sheath
*X. olivaceoconidium*	MFLUCC 15-0380	25–55 × 3–4	11– 15 × 3–5	25–50 × 6–10, 9–16-septate, with a sheath

### ﻿New combinations

#### ﻿ Xenosporidesmium aquaticivaginatum

Taxon classificationFungiDistoseptisporalesXenosporidesmiaceae

(Jing Yang & K.D. Hyde) L. L. Liu & Z. Y. Liu comb. nov.

0866A885-BDDE-5781-AAB1-1F099D2B4546

Index Fungorum: IF904253

≡ Sporidesmium aquaticivaginatum Jing Yang & K.D. Hyde, Fungal Diversity 80: 217 (2016)

##### Material examined.

Thailand • Prachuap Khiri Khan Province, Hua Hin, stream flowing outside Kaeng Krachan National Park, on submerged wood, 25 December 2014, Jaap van Strien, Site4-44-2 (MFLU 15-1159, holotype); ex-type living culture, MFLUCC15-0624; ibid. (HKAS950 46, isotype).

#### ﻿ Xenosporidesmium filiforme

Taxon classificationFungiDistoseptisporalesXenosporidesmiaceae

(W.P. Wang, H.W. Shen, K.D. Hyde & Z.L. Luo) L. L. Liu & Z. Y. Liu comb. nov.

17056FF3-EB18-5D8A-9126-6D98D7F29631

Index Fungorum: IF904254

≡ Sporidesmium filiforme W.P. Wang, H.W. Shen, K.D. Hyde & Z.L. Luo, Mycosphere15(1): 6617 (2024)

##### Material examined.

China • Yunnan Province, Wenshan Zhuang and Miao Autonomous Prefecture, Bamei Town, on submerged decaying wood, 7 February 2022, W.P. Wang, S-3826 (HKAS 130435, holotype).

#### ﻿ Xenosporidesmium guizhouense

Taxon classificationFungiDistoseptisporalesXenosporidesmiaceae

(L. L. Liu & Z. Y. Liu) L. L. Liu & Z. Y. Liu comb. nov.

5AD5E2EF-4EE0-54A2-9C5E-1CFB7B56AE94

Index Fungorum: IF904255

≡ Sporidesmium guizhouense L.L. Liu & Z.Y. Liu, Phytotaxa 422(2): 149 (2019)

##### Material examined.

China • Guizhou Province, Guiyang City, Baihua Lake, 26°39.29'N, 106°32.22'E, on submerged decaying twig, 18 April 2018, Lingling Liu, 18B–40 (HKAS 104644 holotype, GZAAS 19–005 isotype), ex-type living cultures CGMCC 3.19605, GZCC 19–005.

#### ﻿ Xenosporidesmium guttulatum

Taxon classificationFungiDistoseptisporalesXenosporidesmiaceae

(W.P. Wang, H.W. Shen, K.D. Hyde & Z.L. Luo) L. L. Liu & Z. Y. Liu comb. nov.

75E67AEC-61ED-5846-BD27-6A01AC4E5E65

Index Fungorum: IF904256

≡ Sporidesmium guttulatum W.P. Wang, H.W. Shen, K.D. Hyde & Z.L. Luo, Mycosphere 15(1): 6617 (2024)

##### Material examined.

China • Yunnan Province, Wenshan Zhuang and Miao Autonomous Prefecture, Bamei Town, on submerged decaying wood, 7 February 2022, W.P. Wang, S-3481 (HKAS 131397, holotype), ex-type living cultures CGMCC 3.27021 = KUNCC 23–13374.

#### ﻿ Xenosporidesmium nujiangense

Taxon classificationFungiDistoseptisporalesXenosporidesmiaceae

(D.F. Bao, Hong Y. Su, K.D. Hyde & Z.L. Luo) L. L. Liu & Z. Y. Liu comb. nov.

A3F87369-47AB-52E0-9E1E-A09975CC05AC

Index Fungorum: IF904257

≡ Sporidesmium nujiangense D.F. Bao, Hong Y. Su, K.D. Hyde & Z.L. Luo, J. Fungi 7(no. 669): 15 (2021)

##### Material examined.

China • Yunnan Province, Nujiang River, on submerged decaying wood, in July 2016, Z.L. Luo, S983 (HKAS 115795, holotype), ex-type living culture, DLUCC 983.

#### ﻿ Xenosporidesmium olivaceoconidium

Taxon classificationFungiDistoseptisporalesXenosporidesmiaceae

(J. Yang & K.D. Hyde) L. L. Liu & Z. Y. Liucomb. nov.

AEE504DB-53CC-5299-AD59-454C2541716E

Index Fungorum: IF904258

≡ Sporidesmium olivaceoconidium Jing Yang & K.D. Hyde, Fungal Diversity 80: 220 (2016)

##### Material examined.

Thailand • Chiang Rai Province, stream flowing in Tham Luang Nang Non Cave, on submerged wood, 25 November 2014, Jing Yang YJ-14 (MFLU 15-1175, holotype), ex-type living culture, MFLUCC 15-0380, GZCC 16-0008.

## ﻿Discussion

Fresh samples and sequence data are urgently needed to resolve other sporidesmium-like and polyphyletic genera. For example, *Linkosia* was phylogenetically placed in three subclasses (Diaporthomycetidae, Hypocreomycetidae, Sordariomycetidae) in Sordariomycetes (Yang et al. 2018). However, its type species, *Linkosia coccothrinacis* is rarely studied and lacks molecular data, resulting in the natural classification of *Linkosia* unsettled. *Ellisembia* is another sporidesmium-like genus that differs from *Sporidesmium* in having distoseptate conidia (Subramanian 1992). *Ellisembia* was synonymized with *Sporidesmium* by Su et al. (2016), but the same with *Linkosia*, the systematic placement of the type species of *Ellisembia*, *E. coronate* is unknown. Thus, the phylogenetic affinities between *Ellisembia* and *Sporidesmium* need to be further revealed. Due to the largely morphological similarity of *sporidesmium*-like species, we strongly recommend the inclusion of molecular evidence when introducing new taxa into this group to prevent taxonomic confusion in the future.

The Sporidesmiaceae was originally established by Fries (1849), and forms a monophyletic group, currently represented by 20 phylogenetic species (Wang et al. 2024). However, neither the original generic type *Sporidesmium atrum* nor the lectotype *S. ehrenbergii* was included in phylogenetic analyses due to the lack of sequence data. Notably, several sporidesmium-like families exist, such as Distoseptisporaceae (Su et al. 2016; Yang et al. 2021; Zhang et al. 2022; Shen et al. 2024;), Neomassariaceae (Zhang et al. 2024; Xiao et al. 2025; Sun et al. 2025), Pseudosporidesmiaceae (Crous 2008; Crous et al. 2017), and Xenosporidesmiaceae (this study). The family affiliations of *Sporidesmium atrum* and *S. ehrenbergii* need to be further revealed.

## Supplementary Material

XML Treatment for Xenosporidesmiaceae

XML Treatment for Xenosporidesmium

XML Treatment for Xenosporidesmium caohaiense

XML Treatment for Xenosporidesmium aquaticivaginatum

XML Treatment for Xenosporidesmium filiforme

XML Treatment for Xenosporidesmium guizhouense

XML Treatment for Xenosporidesmium guttulatum

XML Treatment for Xenosporidesmium nujiangense

XML Treatment for Xenosporidesmium olivaceoconidium

## ﻿Additional information

### Use of AI

No use of AI was reported.

### Author ORCIDs

Ling-Ling Liu https://orcid.org/0000-0003-0006-0938

Xiao-Feng Gu https://orcid.org/0000-0002-1197-2070

Jiu-Lan Gou https://orcid.org/0000-0001-6439-158X

Zuo-Yi Liu https://orcid.org/0000-0001-5348-8458

Ya-Ya Chen https://orcid.org/0000-0002-8293-168X

Yong-Xiang Liu https://orcid.org/0009-0003-7460-5483

### Data availability

All of the data that support the findings of this study are available in the main text.
